# Emerging Sciences and Innovation in Child and Adolescent Mental Health

**DOI:** 10.1016/j.jaacop.2026.06.002

**Published:** 2026-06-10

**Authors:** Manpreet K. Singh, Robert L. Findling, Mary K. Billingsley, Kara S. Bagot, Joseph Blader, Alice Charach, Timothy E. Wilens, John T. Walkup

## Abstract

Research in child and adolescent mental health stands at an inflection point: the burden of psychiatric illness is global, heterogeneous, and dynamic, whereas our evidence base often remains limited and insufficiently responsive to patient needs. To meaningfully improve outcomes for youth and families globally and at scale, innovation in child and adolescent mental health research must extend beyond developing new treatments to encompass how we design, measure, and disseminate research. Continued innovation is essential to build on the existing corpus of knowledge and to ensure that research keeps pace with real-world clinical and public health priorities.

Research in child and adolescent mental health stands at an inflection point: the burden of psychiatric illness is global, heterogeneous, and dynamic, whereas our evidence base often remains limited and insufficiently responsive to patient needs. To meaningfully improve outcomes for youth and families globally and at scale, innovation in child and adolescent mental health research must extend beyond developing new treatments to encompass how we design, measure, and disseminate research. Continued innovation is essential to build on the existing corpus of knowledge and to ensure that research keeps pace with real-world clinical and public health priorities.

What does innovation look like in this context? It cannot be defined solely by technological novelty or large-scale trials. We must also understand *who needs what treatment, when, and why*, and then deliver those interventions at scale. To accelerate the pace of discovery in these areas, innovation lies in purposeful departures from convention that increase clinical utility, accelerate learning, and better reflect the populations we serve. To that end, *JAACAP Open* is particularly interested in work that is hypothesis generating, methodologically transparent, and immediately informative to clinicians, health systems, and all communities.

Our field must welcome a range of approaches to drive innovation in several key impact areas. We must first move beyond traditional but highly heterogeneous diagnostic categories, toward identifying biologically and clinically meaningful subtypes derived from mechanistic insights using neuroimaging, electrophysiology (electroencephalography), digital phenotyping, genetics, inflammation, reward processing, cognitive control, and sleep and circadian biology over the course of development. In lieu of allocating treatments to patients using trial-and-error approaches, validation of reliable biomarkers should serve to guide treatment selection. Because most major psychiatric disorders begin years before a diagnosis is made, early identification and prevention through risk prediction and preventive trials remain important priorities to achieve the largest population-level gains.

One of the biggest unanswered questions in our field is this: why do psychiatric disorders emerge when they do? Critical sensitive periods likely exist for key neurocognitive processes including emotional regulation, social cognition, reward processing, and executive function, to name a few. Understanding puberty, hormonal transitions, brain maturation, and environmental exposures could reveal entirely new intervention targets. Speaking of targets, neuromodulation and circuit-based treatments are expanding rapidly. Today, circuit-based treatments are expected to demonstrate target engagement, which involves first establishing that a circuit abnormality exists, that intervention changes the circuit, and that the circuit change observed predicts symptom improvement. This increased level of rigor is a departure from simply having a treatment demonstrate symptom improvement alone.

Psychiatric practice still relies heavily on clinic visits and retrospective symptom reporting. However, we anticipate that mental health discovery will continue to include data generated from smartphones, wearables, passive sensing, and ecological momentary assessment to track mood, sleep, activity, social behavior, substance use, and suicide risk in real time. Understanding the interaction between brain health and other physiological systems is a nascent but potentially transformative area, and one in which sensors and smartwatches might come in handy. Some important examples include the interaction between obesity or inflammation and depression, chronic illness and anxiety, sleep disorders and mood, gut–brain interactions, and neuropsychiatric symptoms associated with long COVID. With the emergence and popular use of GLP-1 receptor agonists, for example, some of which are approved for use in youth with diabetes and obesity down to age 12 years, there is a need to understand how this class of drugs affects reward processing, motivation, mood, and suicidality in youth and over time. Given the rapid growth of GLP-1 use in youth, this could become a research focus for many in the short term.

Despite decades of work, suicide remains a leading cause of death among adolescents. The field needs better prediction, as most current risk assessments have limited predictive performance. Researchers can conduct mechanism-focused studies to advance understanding of hopelessness, social isolation, impulsivity, reward dysfunction, sleep disturbance, and emotion dysregulation, or to develop rapid-acting interventions for acute suicidal ideation. Interventions already in development include ketamine, transcranial magnetic stimulation, digital interventions, and intensive prevention models. Even modest advances in suicide prevention could have enormous public health impact, but the challenge is moving beyond prediction to actionable interventions.

These areas all converge on a single goal: shifting child psychiatry from a reactive, symptom-based specialty to a predictive, personalized, and preventive discipline. For investigators across all career stages, the strongest opportunities are often at the intersection of these domains. For example, an investigator might use neuroimaging, digital phenotyping, or physiological biomarkers to identify treatment-responsive subgroups, and then test targeted interventions in prospective trials. These intersections are not only likely to be fundable for many years, but they are also likely to make meaningful impacts.

Other examples of innovation that have potential for impact include small, well-designed trials with high clinical relevance; planned or pre-registered secondary outcomes of clinical interventions that exponentiate the value of already funded or completed studies; and targeted post hoc analyses that advance understanding of which treatments benefit which subgroups of patients or that inform future comparative effectiveness research. These approaches encourage precision medicine, whereby patients can be matched to treatments that are tailored to their individual needs, as in the case of evidence-based medication dosing or measurement-informed care. We also hope that new research in our field will leverage in vivo, real-world data to refine treatment decisions that are free of bias or hallucinations generated from artificial intelligence. Studies that include independent replications or data generated from multiple sites or large or merged databases are critical to demonstrate rigor and to produce findings that are generalizable. We expect research originating from academic, community, or industry settings, but we encourage collaborations among these sectors and diverse stakeholders. Although they are not exhaustive, we believe that any of these approaches can complement established research methods and can provide useful insights for practice, especially when executed with care, rigor, and reproducibility.

Equally important are methodological innovations. As a journal, we aim to serve as a “first-in” youth platform for new approaches, whether in trial design, analytics, or implementation science. Our novel article type “Study Protocol and Methods Advancement” was designed to encourage authors to explicate how their research was breaking new ground. We also believe that pilot studies, early-phase investigations, and novel frameworks are not endpoints but, rather, starting points. Their value lies in their ability to catalyze further inquiry and to shape the next generation of definitive studies.

Our scope is intentionally global and grounded in public health, consistent with the mission of the American Academy of Child and Adolescent Psychiatry, and we recognize that meaningful advances in child mental health will emerge from diverse settings, including low- and middle-income countries. We welcome research that expands our understanding of context, scalability, and equity. Finally, we are committed to timely and accessible dissemination of high-quality research. By sharing well-conducted and controlled studies efficiently, we aspire to encourage the next generation of discoveries to guide progress and to improve outcomes.

We are prepared to take thoughtful risks in publishing emerging work that has the potential to meaningfully move the field forward, even as the evidence base continues to evolve. In this way, *JAACAP Open* aims to function not only as a repository of findings, but as a platform for shaping the trajectory of child and adolescent psychiatry. We invite investigators to share work that is bold in concept, rigorous in execution, and clear in its potential to improve the lives of children and families worldwide.

## CRediT authorship contribution statement

**Manpreet K. Singh:** Conceptualization, Writing – original draft, Writing – review & editing. **Robert L. Findling:** Conceptualization, Writing – original draft, Writing – review & editing. **Mary K. Billingsley:** Conceptualization, Writing – original draft, Writing – review & editing. **Kara S. Bagot:** Conceptualization, Writing – review & editing. **Joseph Blader:** Conceptualization, Writing – review & editing. **Alice Charach:** Conceptualization, Writing – review & editing. **Timothy E. Wilens:** Conceptualization, Writing – review & editing. **John T. Walkup:** Conceptualization, Writing – review & editing.

